# The relationship between parental phubbing and learning burnout of elementary and secondary school students: The mediating roles of parent-child attachment and ego depletion

**DOI:** 10.3389/fpsyg.2022.963492

**Published:** 2022-09-30

**Authors:** Qingqing He, Bihua Zhao, Hua Wei, Feng Huang

**Affiliations:** ^1^School of Educational Science, Anhui Normal University, Wuhu, China; ^2^School of Teacher Education, Hefei Normal University, Hefei, China; ^3^Normal College, Qingdao University, Qingdao, China

**Keywords:** learning burnout, ego depletion, parent–child attachment, elementary and secondary school students, parental phubbing

## Abstract

In this study, we examined the effects of parental phubbing on learning burnout in elementary and secondary school students and its mechanism of action. A questionnaire method was applied to investigate parental phubbing, parent–child attachment, ego depletion, and learning burnout among 2090 elementary and secondary school students in Anhui Province, China. The results are as follows: (1) Parental phubbing was significantly correlated with parent–child attachment, ego depletion, and learning burnout; (2) Parental phubbing has an indirect impact on learning burnout in elementary and secondary school students through three pathways: a separate mediating effect on parent–child attachment, a separate mediating effect on ego depletion, and a chain mediating effect on both. Parental phubbing is a risk factor for Learning Burnout, which can positively affect Learning Burnout in elementary and secondary school students. The findings of the study contribute to revealing the influence mechanism of parental phubbing on learning burnout in elementary and secondary school students.

## Introduction

Learning burnout is a learning-related persistent negative psychological state found in normal individuals (Wu et al., 2007; Steele and Fullagar, 2009), which is manifested in three aspects, including physical and mental exhaustion, academic detachment, and low achievement (Schaufeli et al., 2002; Wu et al., 2010). Research has shown that learning burnout can lead to a series of adverse consequences, including physical dysfunctions such as headache, loss of appetite, joint pain, and general weakness, behavioral problems such as truancy and dropping out of school, and psychological problems such as anxiety and depression (Bask and Salmela-Aro, 2013; Wang et al., 2015). Studies have identified learning burnout in many students and even a trend of spreading to younger students (Wu et al., 2007; Yang et al., 2013; Zhang et al., 2013; Zhu et al., 2021). Previous research results indicate that home environment and parenting style are important factors of learning burnout in elementary and secondary school students (Chang et al., 2015; Luo et al., 2016a,b). Among family factors, the effect of parental phubbing on learning burnout is rarely mentioned. Phubbing, a compound word for phone and snubbing, refers to the behavior of individuals with their heads down and absorbed in their phones in social situations while not minding or even snubbing the people or things around them (Angeluci, 2016; Roberts and David, 2016). Parental phubbing happens when cell phone use occurs in parent–child interactions, where parents focus excessively on their phones and neglect their children instead of caring for or interacting with them (Ding et al., 2018; Xie et al., 2019; Jiang et al., 2021). Previous research has shown that parenting styles with more parental warmth are less likely to induce learning burnout, whereas individuals under parenting styles with more rejections and denials are more vulnerable to learning burnout (Aunola et al., 2000; Li and Gan, 2011; Luo et al., 2016a,b). According to the 2014 National Parent–Child Relationship Report, 17.8% of parents often use their phones, and 51.8% occasionally use their phones when spending time with their children. As a risky environmental factor, parental phubbing may have negative effects on adolescents’ emotions, cognition, learning, and questionable behaviors, thus impairing their learning effectiveness, psychological health, and adaptability development (Reed et al., 2017; Ding et al., 2020). Parental phubbing is a new common parenting behavior. Although previous research has found the negative effects of parental phubbing on learning, its effects on learning burnout have not been explored in depth. Therefore, this study examines the effects of parental phubbing on learning burnout in elementary and secondary school students and its mechanisms of action, which are important for the prevention and intervention of learning burnout in elementary and secondary school students.

### Parental phubbing and learning burnout

Parental phubbing is a new type of neglect and rejection behavior between parents and children (Roberts and David, 2016; David and Roberts, 2017; Allred, 2020). Previous studies found that students whose parents exhibited more neglect and rejection behavior were more prone to learning burnout (Li and Gan, 2011; Shin et al., 2012; Luo et al., 2016a,b; Zhu et al., 2021). And parental phubbing may cause lower satisfaction of children’s basic psychological needs. Self-determination theory (SDT) defines that the basic human psychological needs include autonomy needs, competence needs, and relatedness needs (Deci and Ryan, 1985, 2000). Social situations that satisfy the three psychological needs promote the internalization of external motivation and motivate individuals to persist longer in an activity, thus enabling them to maintain a positive psychological state, grow better, and produce more positive behavioral outcomes. In contrast, environments that impede the satisfaction of these three needs often reduce individuals’ autonomous motivation, work performance, and well-being (Reis et al., 2000; Patrick et al., 2007). The satisfaction of psychological needs is necessary for the optimal development of the individual. Impairing or depriving the satisfaction of any one of the three needs can have significant adverse consequences. Empirical studies have also found that the satisfaction degree of students’ basic psychological needs could effectively predict learning-related behavior outcomes such as learning burnout and academic performance (Jang et al., 2009, 2012), and higher satisfaction degree of the three basic psychological needs means a lower level of learning burnout (Reeve, 2009; Sun and Zhang, 2012; Luo et al., 2014). Therefore, we hypothesized that the level of learning burnout in elementary and secondary school students might increase when perceiving the neglect and rejection from parental phubbing. Based on the above theoretical analysis and empirical results, we propose research hypothesis H1: Parental phubbing is a positive predictor of learning burnout among elementary and secondary school students.

### The mediating role of parent–child attachment

Other than the direct effects of parental phubbing, this study also examines the mediating role of parent–child attachment based on attachment theory. As discussed above, parental phubbing is considered a form of neglect and rejection that can undermine parent–child attachment and affect children’s psychological health development (Xie and Xie, 2020; Wang et al., 2021). Phubbing can cause a sense of social exclusion (David and Roberts, 2017). Adolescents are no exception. They would feel neglected when experiencing parental phubbing. As the earliest form of interpersonal relationship and essentially a relational structure, parent–child attachment is an emotionally enduring bond between parents and children (Ainsworth and Bowlby, 1991). Parental neglect is one of the risk factors affecting parent–child attachment, and children experiencing parental neglect are often reported to have high levels of insecure attachment (Borelli et al., 2015). Therefore, parental phubbing disrupts and reduces the level of parent–child attachment. On the other hand, parent–child attachment has a great impact on the development and adaptation of individuals (Popov and Ilesanmi, 2015). Attachment theory suggests that individuals with secure parent–child attachments are able to fully engage in exploratory activities even facing difficulties due to the protective, supportive, accessible, and empowering roles of the attachment object (usually parents), which ensure that the individuals feel safe and stress-free while engaging in exploratory activities, thereby increasing the willingness and quality of exploration (Bowlby, 1969; Aspelmeier and Kerns, 2003). Empirical studies have also found that the parent–child attachment among family factors is an important factor of learning burnout, i.e., parent–child attachment negatively predicts learning burnout (Zhang, et al., 2019). Improving parent–child communication can reduce learning burnout among secondary school students to some extent (Zhu and Wang, 2009). Factors such as family environment and parenting style affect learning burnout through the mediating effect of parent–child attachment (Wu, 2015). Through the above analysis, this study proposes research hypothesis H2: Parental phubbing affects learning burnout through the mediating effect of parent–child attachment.

### The mediating role of ego depletion

Ego depletion is a significant predictor of learning burnout (Baumeister et al., 1998; Price and Yates, 2010; Seibert et al., 2016). Ego depletion is a temporary decrease in an individual’s ability or willingness to perform volitional activities (Inzlicht and Schmeichel, 2012). The resource model of self-control suggests that controlling attention, emotion regulation, and cognitive processing are all self-control activities that consume the limited self-control resources, which impairs the ability to engage in subsequent self-control tasks, resulting in ego depletion (Inzlicht and Schmeichel, 2012).

Parental phubbing may lead to ego depletion in elementary and secondary school students, which can be explained in cognitive terms. On the one hand, according to expectancy violations theory, individuals always have expectations about the behaviors of each other. During social interactions with others, if the behaviors of others are inconsistent with the individual’s expectations, this expectancy violation can cause arousal and force the individual to make a series of cognitive assessments of the violation (Gong et al., 2019). Phubbing can produce negative expectancy violations (Nakamura, 2015; Gong et al., 2019). Therefore, after sensing the cold shoulder from parental phubbing, which contradicts their expectations of their parents, children would make cognitive assessments of such parental phubbing. For example, they would inquire about the reasons for the frequent parental phubbing. This inconsistency between particular parental behavior and children’s psychological expectations can further increase the cognitive burden and deplete the psychological resources of the children. On the other hand, parental phubbing can induce feelings of rejection in children, triggering a strong claim for attention and belonging (David and Roberts, 2017) and greater sensitivity and attention to parental behavior. Empirical research has found that cognitive processing and attention control greatly deplete self-control resources, thus leading to ego depletion (Inzlicht and Schmeichel, 2012). Based on the above theoretical and empirical evidence, we hypothesize that parental phubbing causes ego depletion. Secondly, ego depletion may lead to learning burnout. In essence, learning burnout is a negative way to cope with learning tasks beyond an individual’s competence (Vasalampi et al., 2009; Tuominen-Soini and Salmela-Aro, 2014; Salmela-Aro et al., 2019), encompassing three dimensions of physical and mental exhaustion, academic detachment, and low achievement. First, ego depletion is sometimes considered a process whereby mental energy is expended during ego activity and takes time to recover afterward, similar to the need for rest to recover from muscle fatigue (Baumeister et al., 1998, 2000; Baumeister, 2000, 2001). In China, elementary and secondary school students learn at a fast pace and under a tight curriculum. With the fatigue from ego depletion, the new learning tasks may lead to physical and mental exhaustion in the learning process. Secondly, individuals have a lower level of attention control when in the ego depletion state (Garrison et al., 2018). Thus, they are easily distracted by other things (Englert et al., 2015), which is not conducive to the completion of learning tasks. In addition, construal level theory suggests that individuals in a state of ego depletion focus more on the achievability of events or tasks, especially the difficulty and completion method. Thus, they are more likely to choose tasks that are easier to achieve but less valuable (Fujita, 2008). For students, the achievable tasks in a state of ego depletion may include recreational activities other than academics or other tasks that appear easier than the academic work due, all of which can contribute to academic detachment. Finally, ego depletion may be followed by cognitive biases manifesting as an underestimation of one’s own capabilities, a negative assessment of one’s own control over the external environment, and more pessimistic expectations for the future (Fischer et al., 2007). This can decrease an individual’s learning self-efficacy and cause a low sense of accomplishment. Empirical studies have also found that self-efficacy is an effective predictor of learning burnout (Zhu and Wang, 2009). With the above analysis, we propose research hypothesis H3 based on the ego depletion theory: Parental phubbing significantly and positively predicts the level of learning burnout in elementary and secondary school students through the mediating effect of ego depletion.

### The chain mediating roles of parent–child attachment and ego depletion

In addition to examining the isolated mediating roles of parent–child attachment and ego depletion, we also examined their chain mediating roles based on parental acceptance-rejection theory. Parental acceptance-rejection theory suggests that parenting includes the dimensions of acceptance and rejection, where the acceptance and inclusion from parents promote the healthy development of children while parental neglect and rejection negatively affect the cognitive, behavioral, and emotional development of adolescents (Rohner, 2004; Fard et al., 2015). Parental neglect and rejection can also have a range of negative effects on children and adolescents, e.g., low parent–child attachment, ego depletion, and learning burnout (Shin et al., 2012). Nevertheless, the relationship between the outcomes induced by parental refusal behaviors is not clear. This study attempts an extension of the parental acceptance-rejection theory to examine whether parental rejection behaviors (parental phubbing) in the Internet era affect the levels of learning burnout in elementary and secondary school students through the chain mediating effects of parent–child attachment and ego depletion. According to the attachment theory, the mental representations and cognitive patterns of self and others affect an individual’s processing of knowledge experience and emotional affect in the self and social systems (Bowlby, 1969). Secure attachment provides an individual with an “internal working model” to confidently explore the external world. This “internal working model” helps individuals develop a healthy schema to positively understand the intentions of others. Individuals with low parent–child attachment tend to have higher levels of negative emotions such as depression and anxiety (Peter, 2000; Ju and Lee, 2017; Lin et al., 2018). According to the ego depletion theory, the self-regulation of negative emotions consumes many self-control resources and weakens self-control (Baumeister et al., 2007), and negative emotions also lead to higher levels of ego depletion (Inzlicht and Schmeichel, 2012). Ego depletion is the decrease in cognitive levels and executive function due to the consumption of psychological energy (Schmeichel et al., 2003; Tan et al., 2012), which negatively impacts academic success (Price and Yates, 2010). Through the above analysis, we propose research hypothesis H4: parental phubbing affects learning burnout in elementary and secondary school students through the chain mediating effects of parent–child attachment and ego depletion.

### Hypotheses of this study

**Figure 1:**
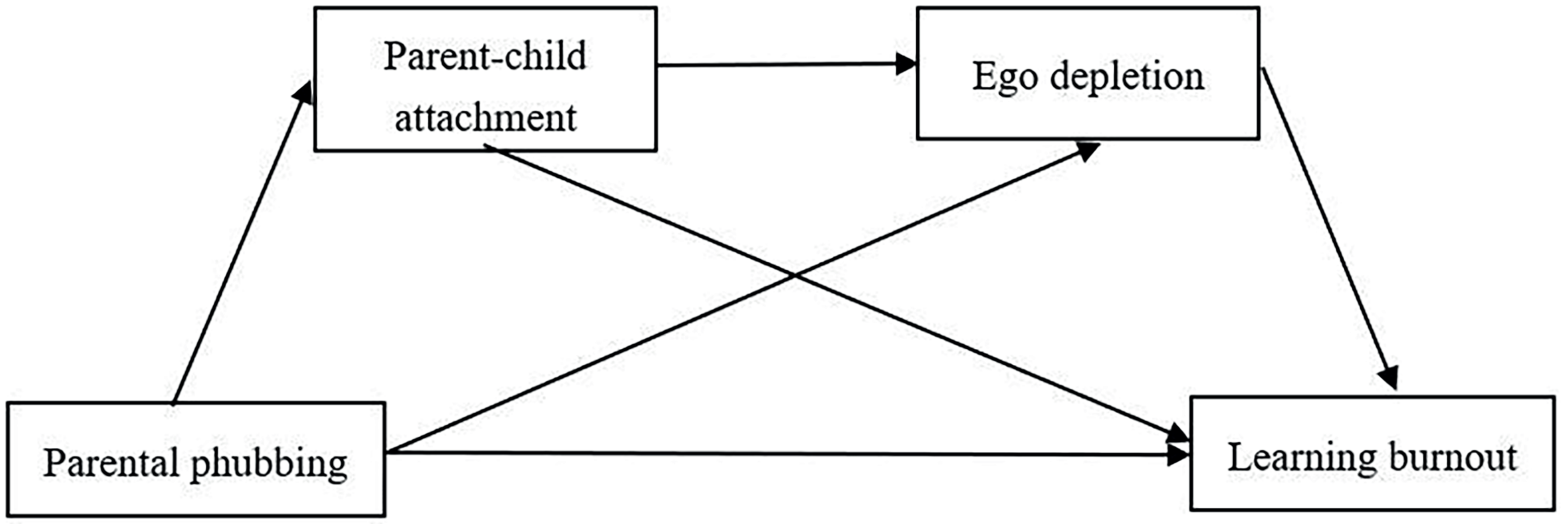
The proposed mediation model.

In this study, a serial mediation model (Figure 1) was proposed to test the mediating role of parent–child attachment and ego depletion in the association between parental phubbing and learning burnout in elementary and secondary school students. Based on reviews of the relevant.

studies, we had the following tentative hypotheses.

*Hypothesis 1*: Parental phubbing is positively associated with learning burnout in elementary and secondary school students.

*Hypothesis 2*: Parent–child attachment plays a mediating role between parental phubbing and learning burnout in elementary and secondary school students.

*Hypothesis 3*: Ego depletion plays a mediating role between parental phubbing and learning burnout in elementary and secondary school students.

*Hypothesis 4*: Parent–child attachment and ego depletion play a chain mediating role between parental phubbing and learning burnout in elementary and secondary school students.

## Materials and methods

### Participants and procedure

Through random cluster sampling, 2,090 students in grades 4 to 9 were selected from two elementary schools and two secondary schools in Anhui Province, China. After excluding the invalid questionnaires with missing answers or consistent responses, 1,967 valid questionnaires were recovered, with an effective rate of 94%. Among them, 1,001 of the valid respondents were boys, and 966 were girls; the valid respondents included 273 in the fourth grade, 243 in the fifth grade, 252 in the sixth grade, 340 in the seventh grade, 427 in the eighth grade, and 432 in the ninth grade. The minimum age of the respondents was 9 years, and the maximum age was 17 years, with a mean age of 12.26 years and a standard deviation of 1.74 years.

### Instruments

#### Parental phubbing scale

A revised Parental Phubbing Scale (Ding et al., 2020) was adopted, which was single-dimensional with nine items, such as “My parents do not use their phone when we are talking.” The items were rated on a 5-point Likert scale from “Never” to “Always.” The higher the total score, the more intensive the parents focus on their cell phones while neglecting or snubbing their children. In the present study, the coefficient α of this scale is 0.73.

#### Ego depletion scale

A simplified Ego Depletion Scale was applied (Lanaj et al., 2014), which had five items, such as “I feel exhausted.” The 7-point Likert scale ranging from “Strongly disagree” to “Strongly agree” was adopted, where higher scores indicate higher degrees of ego depletion. The scale is suitable for the Chinese version with good reliability and validity (Ding et al., 2020). In this study, the internal consistency coefficient α of the scale is 0.80.

#### Parent–child attachment scale

A revised version of the Inventory of Parent and Peer Attachment (IPPA-R) was used to measure the respondents’ attachment to their parents (Chen et al., 2015). The questionnaire had 13 items, such as “My parents respect my feelings.” A 5-point Likert scale ranging from “Never” to “Always” was adopted. Higher scores indicate higher levels of parent–child attachment. In this study, the internal consistency coefficient α of the scale is 0.81.

#### Adolescent learning burnout scale

The Adolescent Learning Burnout Scale developed by Wu et al. (2010) was adopted, which included dimensions such as physical and mental exhaustion, academic alienation, and low achievement. Its 16 items included “I feel so empty recently, I do not know what to do,” and “I am so bad at studying and really want to give up.” A 5-point Likert scale ranging from “Strongly disagreeable” to “Strongly agreeable” was used, with higher total scores indicating higher levels of learning burnout. In this study, the coefficient α of this scale is 0.80.

### Statistical analysis

In this study, trained postgraduates majoring in psychology conducted the test on a class basis, and the questionnaires were distributed and collected on the spot. Descriptive statistics and correlation analysis were carried out on SPSS 18.0. The chain mediation model tests were performed using the SPSS macro programs PROCESS MODEL 6 compiled by Hayes. Significance testing of regression coefficients was performed using Bootstrap (sampling repeated 5,000 times) to obtain robust standard errors and a 95% bias-corrected confidence interval (CI) for parameter estimation. In addition, age and gender were included as control variables. The Harman single-factor test method was applied to process all measurement items through nonrotating exploratory factor analysis. According to the analytical results, eight common factors with eigenvalues greater than 1 were extracted, the first of which explained 20.15% of the total change, thus falling short of the 40% standard threshold. That is, no deviation is caused by the same data collection method in this study (Podsakoff et al., 2003).

## Results

### Descriptive statistics and correlation analysis

According to correlation analysis, parental phubbing is significantly and positively correlated with learning burnout; ego depletion is significantly and positively correlated with parental phubbing and learning burnout; and parent–child attachment is significantly and negatively correlated with parental phubbing and learning burnout (Table 1).

**Table 1:** Mean, standard deviation, and correlation matrix of each variable.

Variables	M	SD	1	2	3	4	5	6
1. Gender	0.51	0.50	1					
2. Age	12.26	1.73	−0.02	1				
3. Parental phubbing	2.57	0.72	−0.11^***^	0.17^***^	1			
4. Parent–child attachment	3.55	0.89	0.04	−0.16^***^	−0.38^***^	1		
5. Ego depletion	2.07	0.78	−0.05^*^	0.15^***^	0.25^***^	−0.38^***^	1	
6. Learning burnout	2.61	0.69	−0.08^***^	0.19^***^	0.25^***^	−0.46^***^	0.61^***^	1

*p < 0.05; **p < 0.001.

### Parental phubbing and learning burnout: Chain mediating effect test

**Figure 2:**
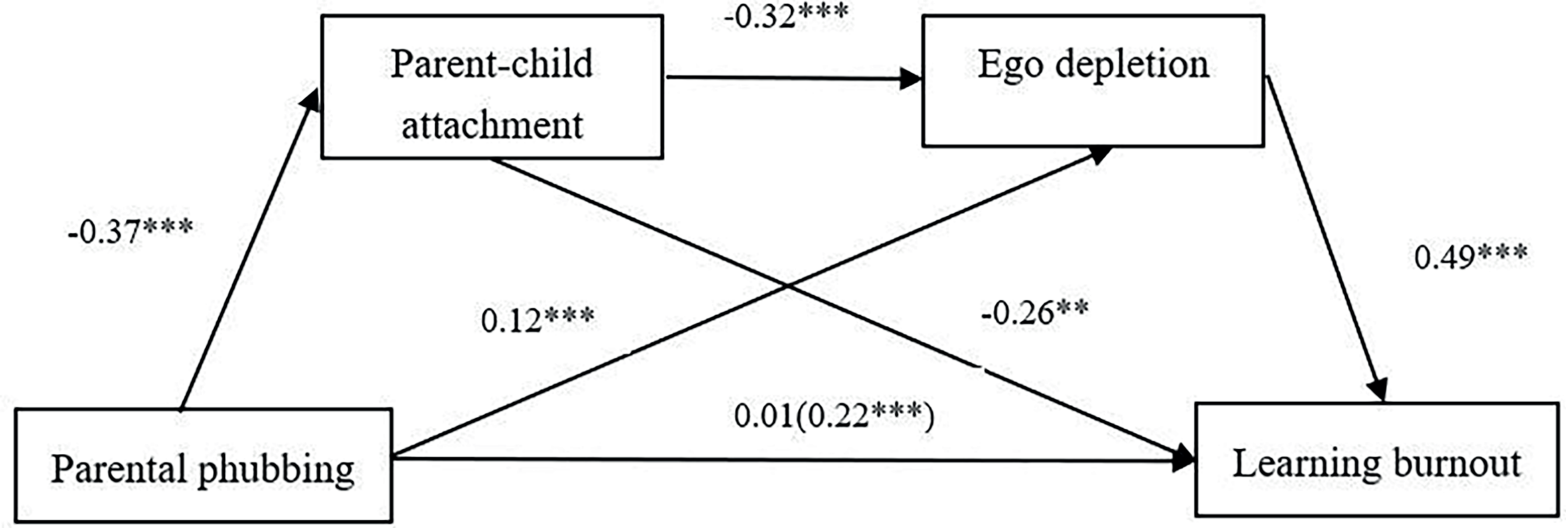
Serial mediation model shows effects of Parental phubbing, Parent–child attachment, and Ego depletion on Learning burnout. *N* = 1,967. The total effect of Parental phubbing is shown in parentheses. Regression coefficients were obtained after controlling for age and sex in PROCESS Procedure for SPSS. ^***^p < 0.001.

A chain mediation model was tested, which consisted of three indirect effects as follows: (1) parental phubbing promotes learning burnout *via* parent–child attachment, (2) parental phubbing promotes learning burnout *via* ego depletion, and (3) parental phubbing promotes learning burnout *via* parent–child attachment and ego depletion (Figure 2).

After controlling the effects of age and gender, the results showed a positive effect of parental phubbing on parent–child attachment, *B* = −0.37, *t* = −17.02, *p* < 0.001, and a positive effect of parental phubbing on ego depletion, *B* = 0.12, *t* = 5.05, *p* < 0.001. A negative relationship between parent–child attachment and ego depletion was also identified, *B* = −0.32, *t* = −14.53, *p* < 0.001. Moreover, parent–child attachment significantly predicted learning burnout, *B* = −0.26, *t* = −13.38, *p* < 0.001. Ego depletion significantly predicted learning burnout, *B* = 0.49, *t* = 26.61, *p* < 0.001. The total effect of parental phubbing on learning burnout was statistically significant, *B* = 0.22, *t* = 9.84, *p* < 0.001. After controlling the effects of parent–child attachment, ego depletion, age, and gender, the direct effect of parental phubbing on learning burnout was not significant, *B* = 0.01, *t* = 0.49, *p* = 0.63 > 0.01.

Furthermore, the indirect effect of parental phubbing on learning burnout through parent–child attachment was significant, *B* = 0.095, *SE* = 0.011, 95% CI [0.073, 0.117]. The mediation effect (parental phubbing → parent–child attachment → learning burnout) accounted for 43.18% of the total effect. Also, ego depletion mediated the relationship between parental phubbing and learning burnout, *B* = 0.057, *SE* = 0.013, 95% CI [0.031, 0.083]. The mediation effect (parental phubbing → ego depletion → learning burnout) accounted for 25.91% of the total effect. Finally, the indirect effect of parental phubbing on learning burnout through parent–child attachment and then ego depletion (i.e., a chain mediating effect) was also found, *B* = 0.059, *SE* = 0.008, 95% CI [0.043, 0.076]. The mediation effect (parental phubbing → parent–child attachment → ego depletion → learning burnout) accounted for 26.82% of the total effect. The direct and indirect effects of parent–child attachment and ego depletion on the relationship between parental phubbing and learning burnout are shown in Table 2. Since 0 is not contained in the Bootstrap 95% confidence intervals, these three indirect effects are statistically significant.

**Table 2:** Direct, indirect, and total effects of parental phubbing on learning burnout.

Model pathways	Estimated effect (*β*)	95%CI	
		Lower	upper
**DIRECT EFFECT**			
PH → LB	0.009	−0.029	0.046
**INDIRECT EFFECTS**			
PH → PCA → LB	0.095^**^	0.073	0.117
PH → ED → LB	0.057^**^	0.031	0.083
PH → PCA → ED → LB	0.059^**^	0.043	0.076
Total effect	0.219^***^		

PH, parental phubbing; PCA, parent–child attachment; ED, ego depletion; LB, learning burnout.

** *p* < 0.01;

*** *p* < 0.001.

#### Discussion

### Parental phubbing and learning burnout

This study found that parental phubbing is significantly related to learning burnout in elementary and secondary school students, which is consistent with the research on parental phubbing and the internalizing and externalizing problems in children and adolescents. Thus, the negative effects of parental phubbing on child and adolescent development are consistent, which include internalizing problems such as anxiety and depression and externalizing problems such as reduced learning efficiency, Internet addiction, and aggressive behavior (Sharaievska and Stodolska, 2016; Cho and Lee, 2017; Reed et al., 2017; Stockdale et al., 2018; Xie et al., 2019). Healthy socio-emotional development in children depends on sensitive parent–child interactions (Kelly et al., 2011). Parental phubbing during parent–child interaction makes it difficult for parents to identify and respond to children’s individual needs in time, resulting in problem behaviors in children(Jiang et al., 2021). That also proves that individuals who grow up under the rejection parenting style experience more frustrations, feel more pressure and helplessness, and then experience academic burnout (Shin et al., 2012; Luo et al., 2016a,b). However, the present study is the first to discuss the impact of parental phubbing on learning burnout. Learning burnout has significant impacts on learning and life. It can lead to a range of adverse developmental outcomes, such as low levels of academic achievement, truancy, absenteeism, dropping out of school, and even psychological problems and disorders (Bask and Salmela-Aro, 2013; Wang et al., 2015). This is especially true in Chinese society, where learning is highly valued, and academic performance is considered highly important by parents. However, parental phubbing is becoming increasingly common with the popularity of smartphones, and many parents neglect the impact of phubbing on their children’s learning. The results of this study have practical implications in that it suggests that parental phubbing is an important factor inducing learning burnout in elementary and secondary school students.

### The mediating role of parent–child attachment

Secondly, this study found that parent–child attachment mediated the relationship between parental phubbing and learning burnout, i.e., parental phubbing affected the learning burnout in elementary and secondary school students by decreasing parent–child attachment levels. This finding is consistent with the findings of previous studies on how parental phubbing adversely affects adolescents through parent–child attachment, e.g., Internet addiction (Cho and Lee, 2017; Xie et al., 2019; Zhang et al., 2021). Thus, the internal mechanisms by which parental phubbing affects questionable behaviors in adolescents are consistent. As an extension of previous research, this study suggests that parental phubbing impacts the learning problems in elementary and secondary school students through parent–child attachment. This suggests that parental phubbing is an important parenting behavior. According to parental acceptance-rejection theory, parental acceptance provides a warm home environment that promotes parent–child attachment and facilitates the children to explore with greater willingness. Parental phubbing is considered a rejection behavior and negative parenting behavior. Positive parent–child relationships enhance adolescents’ ability to cope with academic stress and their motivation, initiative, and persistence in learning, thus effectively curbing learning burnout (Luo et al., 2016a,b).

### The mediating role of ego depletion

This study also identified the mediating effect of ego depletion between parental phubbing and learning burnout, i.e., parental phubbing increases the risk of learning burnout in elementary and secondary school students by affecting ego depletion. This result is consistent with the Chinese and international research findings that higher levels of ego depletion are associated with higher levels of learning burnout (Price and Yates, 2010; Zhang, 2010), suggesting the important mediating role of ego depletion in the process of parental phubbing affecting the learning burnout in elementary and secondary school students. Parental phubbing is considered a new form of neglect and rejection (Roberts and David, 2016; David and Roberts, 2017; Allred, 2020). According to limited self-control theory, individuals consume part of their limited cognitive resources after experiencing social exclusion, which reduces their level of self-control and induces ego depletion. As a typical manifestation of poor interpersonal relationships, social exclusion affects ego depletion (Baumeister et al., 2005; Bertrams and Pahl, 2014). Ego depletion is a process by which activities originating from the ego deplete mental energy and cause a decline in executive function (Tan et al., 2012). As a social exclusion behavior, parental phubbing depletes self-control resources and results in ego depletion, which in turn leads to reduced self-regulation, difficulties in emotion control, difficulties in attention control, and stress (Tan et al., 2012). These, in turn, increase the risk of learning burnout in elementary and secondary school students. In addition, previous studies of social exclusion leading to ego depletion have generally adopted the experimental paradigms of social exclusion induction, such as the accidental exclusion paradigm and the banishment paradigm (Williams and Jarvis, 2006; Park and Baumeister, 2015). This study is an expansion of the social exclusion research context by examining social exclusion (parental phubbing) in a real-life context.

### The chain mediating roles of parent–child attachment and ego depletion

Overall, this study identified the chain mediating effects of parent–child attachment and ego depletion between parental phubbing and learning burnout, suggesting that elementary and secondary school students perceiving more parental phubbing have elevated levels of ego depletion and ultimately suffer from learning burnout. On the one hand, parent–child attachment was found to be an important mediator in explaining the effect of parental phubbing on children’s adaptability, which is consistent with previous findings (Xie et al., 2019; Niu et al., 2020). However, few previous studies have continued to explore the mediating processes by which parent–child attachment affects children’s adaptability. This study expands one mediating factor from previous studies into an intermediary chain. The explanatory mechanism of parental phubbing affecting children’s academic adaptability was enriched from the perspective of ego depletion theory. Since self-control resources can be restored after ego depletion (Baumeister, 2000), interventions such as positive emotion induction (Tice et al., 2007) become possible, which are useful advances to previous research. In addition, the results support the parental acceptance-rejection theory, suggesting that parenting styles of rejection and neglect can negatively affect children and adolescents (Rohner et al., 2010). Moreover, compared with the rough parenting and parental neglect in previous studies (Shin et al., 2012; Qi et al., 2020), the parental phubbing explored in this study is a more insidious factor. This result also enriches the outreach of parental acceptance-rejection theory and expands its scope.

### Practical significance

The results of this study have important implications for the prevention and intervention of learning burnout in secondary school students. For one thing, parents are an important part of the family system, and their parenting style plays an important role in children’s cognition, emotion, behavior, attitude, academic performance, and even personality building (Jones et al., 2012). Parental neglect and rejection cause the children to be more prone to negative emotions and learning burnout (Blondal and Adalbjarnardottir, 2014; Waterman and Lefkowitz, 2017). Parents should be reminded as much as possible to be aware of what they say and do in their lives to prevent the creation of a risky ecological environment, e.g., avoiding excessive phubbing during parent–child communication. For another, the chain mediating effect suggests that parental phubbing affects learning burnout in elementary and secondary school students through interpersonal and individual factors. Thus, interventions can also be designed based on family and individual factors. For example, parents should actively maintain parent–child relationships, put down their cell phones to communicate more with their children, and jointly develop cell phone use norms; children should actively participate in group activities at school, learn to timely confide in their classmates or friends, and actively express their inner needs to their parents; In the meantime, positive emotional guidance and other methods should be employed to reduce the ego depletion caused by parental phubbing. This also indicates that taking an integrated perspective on learning burnout in elementary and secondary school students and integrating family and individual factors into the microenvironmental systems of child and adolescent development can help improve the effectiveness of interventions.

## Limitations and future orientation

Some aspects of this study still need attention and improvements. Firstly, as a cross-sectional study, this research investigated the effects of parental phubbing on learning burnout in elementary and secondary school students but could not determine the causal relationships and developmental changes. Further in-depth studies could be conducted in the future through longitudinal follow-up. Secondly, this study examined parental phubbing as a whole and did not distinguish between paternal and maternal phubbing, while previous studies have shown the different effects of paternal and maternal behaviors of electronics usage on their children (Mcdaniel and Radesky, 2018). Thirdly, other studies in this field noted a bidirectional effect of parental phubbing (Gong et al., 2019). Specifically, a phubbing individual neglects others, and this attitude, in turn, promotes the individual’s dependence on electronics. The special affections of parents for their children are different from other interpersonal relationships, and whether this bidirectional effect still exists is uncertain. If this bidirectional effect exists, the extent to which such a vicious cycle affects adolescents requires investigation.

## Conclusion

In summary, this study found that: (1) Parental phubbing was significantly negatively associated with parent–child attachment and significantly positively associated with ego depletion and learning burnout. Parent–child attachment was significantly and negatively associated with ego depletion and learning burnout. Ego depletion was significantly and positively associated with learning burnout. (2) Parental phubbing affects learning burnout through the isolated mediating effect of parent–child attachment; Parental phubbing affects learning burnout through the isolated mediating effect of ego depletion; and Parental phubbing affects learning burnout through the chain mediating effects of parent–child attachment and ego depletion.

## Data availability statement

The raw data supporting the conclusions of this article will be made available by the authors, without undue reservation.

## Ethics statement

The studies involving human participants were reviewed and approved by the Ethics Committee of Anhui Normal University. Written informed consent to participate in this study was provided by the participants’ legal guardian/next of kin.

## Author contributions

QH and BZ contributed to the conception and design of the study. QH organized the database and wrote the first draft of the manuscript. BZ wrote sections of the manuscript. HW and FH performed the statistical analysis. All authors contributed to the article and approved the submitted version.

## Funding

The study was financially supported by the Philosophy and Social Science Planning Project of Anhui Province of China (AHSKZ2021D11).

## Conflict of interest

The authors declare that the research was conducted in the absence of any commercial or financial relationships that could be construed as a potential conflict of interest.

## Publisher’s note

All claims expressed in this article are solely those of the authors and do not necessarily represent those of their affiliated organizations, or those of the publisher, the editors and the reviewers. Any product that may be evaluated in this article, or claim that may be made by its manufacturer, is not guaranteed or endorsed by the publisher.
